# *In Vivo* Cell and Tissue Dynamics Underlying Zebrafish Fin Fold Regeneration

**DOI:** 10.1371/journal.pone.0051766

**Published:** 2012-12-20

**Authors:** Rita Mateus, Telmo Pereira, Sara Sousa, Joana Esteves de Lima, Susana Pascoal, Leonor Saúde, Antonio Jacinto

**Affiliations:** 1 Instituto de Medicina Molecular, Faculdade de Medicina de Lisboa, Lisboa, Portugal; 2 PhD Programme in Experimental Biology and Biomedicine (5th PDBEB), Center for Neuroscience and Cell Biology, University of Coimbra, Coimbra, Portugal; 3 Instituto Gulbenkian Ciência, Oeiras, Portugal; 4 Centro de Estudos de Doenças Crónicas, Faculdade de Ciências Médicas, Campo Mártires da Pátria, Lisboa, Portugal; Institute of Science and Technology Austria, Austria

## Abstract

**Background:**

Zebrafish (*Danio rerio*) has a remarkable capacity to regenerate many organs and tissues. During larval stages the fin fold allows the possibility of performing long time-lapse imaging making this system very appealing to study the relationships between tissue movements, cell migration and proliferation necessary for the regeneration process.

**Results:**

Through the combined use of transgenic fluorescently-labeled animals and confocal microscopy imaging, we characterized *in vivo* the complete fin fold regeneration process. We show, for the first time, that there is an increase in the global rate of epidermal growth as a response to tissue loss. Also enhanced significantly is cell proliferation, which upon amputation happens in a broad area concerning the amputation level and not in a blastema-restricted way. This reveals a striking difference with regard to the adult fin regeneration system. Finally, an accumulation of migratory, shape-changing fibroblasts occurs proximally to the wound area, resembling a blastemal-like structure, which may act as a signaling center for the regeneration process to proceed.

**Conclusions:**

These findings provide a novel *in vivo* description of fundamental mechanisms occurring during the fin fold regeneration process, thereby contributing to a better knowledge of this regenerative system and to reveal variations in the epimorphic regeneration field.

## Introduction

Most vertebrates, including humans, are unable to regenerate the majority of lost or damaged tissues. In contrast, lower vertebrates such as zebrafish (*Danio rerio*) are able to regenerate many of their organs upon amputation. These animals have the amazing and extremely useful capacity of restoring a fully functional organ, through a process of epimorphic regeneration. This type of regeneration involves a specialized and transient tissue, the blastema, that plays a key role in the signaling and proliferation events that are necessary to recover the lost organ [1]. The blastema is composed of undifferentiated cells that are recruited to the amputation plane [2] [3] [4] [5], and is enclosed by a wound epidermis that also plays a central role in terms of signaling [6] [7] [8].

**Figure 1:**
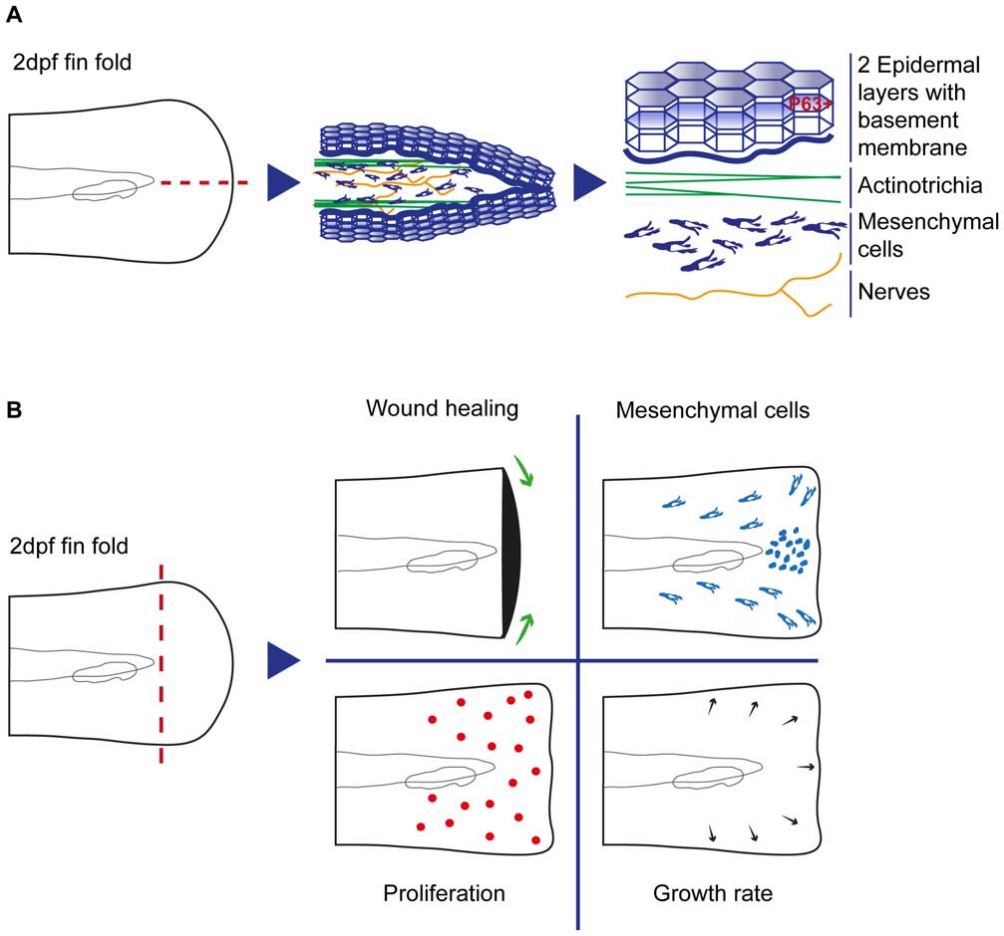
Fin fold organization and regeneration. **A** Representation of the composition of the 2 dpf fin fold cell types and structures, including their respective organization. **B** Schematic overview representing the main findings occurring after amputation of the 2 dpf larva fin fold, as a part of the regenerative process, addressed throughout the manuscript.

The zebrafish has emerged as a powerful model system to perform regeneration studies due to its high regenerative potential coupled to availability of genetic tools. Kawakami *et al* (2004) proposed a new zebrafish-based regeneration system: the early fin primordium (or fin fold) of the 2 days post-fertilization (dpf) larva [9]. This model was established on the basis of its similarities to the adult zebrafish caudal fin system. In particular, the existence of the three regeneration phases (wound healing, blastema formation and regenerative outgrowth), the formation of similar structures upon amputation (i.e. wound epidermis), and a large number of coincident upregulated expression markers [10] [11]. In addition, the fin fold model presents some advantages in comparison to the adult model, namely the speed of regeneration, since in the fin fold the full process takes only 72 hours to complete restoration of the lost tissue, and the structural simplicity of this non-vascularized appendage [12] since it is only composed of five layers of tissue. In the larva fin fold, a middle layer of mesenchyme, composed of fibroblast-like cells [13] [14], nerves and actinotrichia [15] [16], is surrounded by two layers of epidermis containing basal p63-positive keratinocytes, with underlying basement membranes [17] [18] (Fig. 1A).

Our goal was to characterize *in vivo* the complete fin fold regeneration process, by using advanced time-lapse confocal imaging of transgenic animals. In particular, we followed the three regeneration stages to unveil how the tissue behaves and recovers after an amputation, in terms of interactions between epidermal layers of tissue and individual migratory mesenchymal cells. Furthermore, we analyzed the orientation of cell division and rate of proliferation in a systematic manner. We show that there is an increase in the global rate of epidermal growth as a response to tissue loss that is not directly dependent on local proliferation. Interestingly, proliferation is enhanced upon amputation but happens in a broad area surrounding the amputation level and not in a blastema-restricted way. This reveals a striking difference with regard to the adult system. Additionally, we found that a population of polarized, migratory, shape-changing mesenchymal cells accumulates proximally to the wound area, resembling a blastemal-like structure, which may act as a signaling center for the regenerative process (Process Overview Fig. 1B).

## Materials and Methods

### Ethics Statement

All experiments involving animals were approved by the Animal User and Ethical Committees at Instituto de Medicina Molecular, according with directives from Direcção Geral Veterinária (PORT 1005/92).

### Zebrafish lines, maintenance and surgery

All Zebrafish lines used were maintained in a re-circulating system with a 14 h/day and 10 h/night cycle at 28°C. Embryos were gathered as described in *The Zebrafish Book* and kept in E3 zebrafish embryo medium at 28°C until reaching the desired developmental stage. Both AB and Tuebingen wild-type lines were used. The transgenic lines used for live imaging were: Tg(*β-actin:mGFP*) [19], Tg(*H2a.f/z-GFP*)^kca66^ [20], Tg(*actb1:myl12.1-eGFP*) [21], Tg(*EF1α:mKO2-zCdt1(1/190)*)^rw0405b^ [22] and GT(*ctnna-Citrine*)^ct3a^ [23]. These lines were kindly provided by Mathias Koppen, Zirc, Carl-Philipp Heisenberg, Atsushi Miyawaki and Mihaela Zigman, respectively. All fin fold amputations were performed in embryos anaesthetized in 0.1% MS-222 (Sigma) using a scalpel as previously described [9]. Regeneration was then allowed to proceed until defined time points at 28°C.

### *osteopontin:eGFP* transgenic line generation

A bacterial artificial chromosome (BAC) that included the zebrafish *osteopontin* (also known as *spp1*) locus (CH73-213K3, BACPAC Resources Center) was used as a template to amplify a 1295 bp fragment, comprising the 1244 bp sequence upstream of the translational start site and the first 51 bp of the coding sequence. The following set of primers were used for amplification: Fwd 5′CATGATATCTCAGGGCACTACGG3′ and Rev 5′TACACAGAAGACTGTGGCGACG3′. The promoter region was cloned in a modified version of pMinitol:MCS [24] that included *βglobin-5′HS4* insulator sequences flanking the transgene [25]. The details on the cloning protocol can be provided upon request. Microinjections to generate the transgenic embryos were performed at one cell-stage of wild-type AB strain, according to standard procedures. The final plasmid was named *pMinitol2.5-osteopontin:eGFP* and 52 ng/µL of DNA was co-injected with 112 ng/µL of capped transposase mRNA and 1% of rhodamine B dextran (10,000 MW, Invitrogen), diluted in 1× Danieau's solution (58 mM NaCl, 0.7 mM KCl, 0.4 mM MgSO_4_, 5 mM HEPES, 0.6 mM Ca(NO_3_)_2_).

### Microinjection of zebrafish embryos

Wild-type AB strain one-cell stage embryos were injected using standard procedures with 100 pg Utrophin-GFP mRNA, produced by linearization of pcs2-utrophin-GFP [26] with NotI (Fermentas), and transcribed using the SP6 mMESSAGE mMACHINE High Yield Capped RNA Transcription Kit (Ambion). A PV-820 Pico-injector (World Precision Instruments) and a Narashige micromanipulator were used for microinjection.

### Live imaging

Wound healing time-lapse imaging was performed in 2 dpf injected Utrophin-GFP, *actb1:myl12.1-eGFP* or *ctnna-Citrine* transgenic embryos. Animals were amputated 5 minutes prior to imaging. Sequential time-lapse imaging was performed in *β-actin:mGFP* and *H2a.f/z-GFP* 2 dpf, 3 dpf and 4 dpf embryos both in uncut and amputated fin folds at several regeneration stages. In both cases, a Zeiss5Live confocal microscope was used and images were acquired using a 20× dipping objective every minute for wound healing imaging, every 2 minutes for *β-actin:mGFP* experiments, and every 10 minutes for *H2a.f/z-GFP* experiments. Long time-lapse imaging (up to 12 h) and shape monitoring time-lapse imaging (up to 3 h) was performed using 2 dpf double positive embryos from an *osteopontin:eGFP* and *EF1α:mKO2-zCdt1* cross both in uncut and amputated fin folds. Images were acquired every 5 minutes for long time-lapse and every minute for shape monitoring time-lapse, using a ZeissLSM710 with a 40× oil objective. All *in vivo* imaging was performed in anaesthetized animals with 0.1% MS222 (Sigma) diluted in E3 zebrafish embryo medium.

### Immunofluorescence

This protocol was adapted from [27] with the following modifications: embryos were fixed in 4% paraformaldehyde (PFA) (Sigma) at 4°C overnight (o/n), then transferred to 100% Methanol (MeOH) (Merck) and stored at −20°C o/n. Then embryos were rehydrated gradually in series of MeOH/phosphate-buffered saline (PBS) and washed twice for 5 minutes with 1% PBS-TritonX-100, followed by a 7 minute permeabilization with 100% acetone at −20°C. Then the embryos were washed in 0.1% Triton-X100, 1% DMSO (Sigma) and 1% Bovine Serum Albumin (BSA) (Sigma) in PBS (PBDX), followed by a 2 hour blocking in 0.1% Triton-X100, 1% DMSO, 1% BSA and 5% goat serum in PBS. The embryos were incubated in primary antibodies (Anti-GFP rabbit, 1∶100, Invitrogen; anti-γ Tubulin mouse, 1∶200, Sigma; anti-active Caspase 3 rabbit, 1∶400, AbCam) diluted in blocking solution, o/n at 4°C. The embryos were then washed several times in PBDX and incubated in secondary antibodies (1∶500, Alexa Fluor 488 anti-rabbit, Alexa Fluor 568 anti-mouse, Invitrogen) and/or with phalloidin (1∶200, conjugated with Alexa Fluor 568, Invitrogen) diluted in blocking solution, o/n at 4°C. The next day, embryos were washed several times as previously and DAPI (Sigma) was applied in 0.001 mg/mL of PBS. Embryos were mounted in 80% Glycerol, 2% DABCO (Sigma) diluted in PBS and then imaged using a ZeissLSM710 confocal microscope. In stainings where phalloidin was used, there was no MeOH transfer and there was a direct continuation of the protocol after o/n fixation with PFA.

### 3D Image processing

To create the 3D images, the original z-stack acquired data was treated using the Imaris software with the *easy 3D* option.

### Image analysis and quantification

For all movie analysis, maximum intensity z-stack projections were made using the LSM Image Browser software.

For tissue movement analysis, raw images were registered using the ImageJ plugin StackReg (rigidbody transformation), to correct for non-specific movement. Vector velocity fields were obtained using mpiv toolbox for Matlab (64 pixel window, 0.5 window overlap, 40 pixel window displacement, mqd algorithm, with two recursive checks). Resultant vector fields were filtered (median filter, threshold 2, kriging interpolation) and smoothed (weighting method) to remove stray vectors and homogenize the result respectively. This analysis was performed using three images, equally separated in time (2.5 hours), for each time-lapse movie. To remove erratic vectors which appear outside the fin fold area, images were segmented using the Matlab watershed algorithm (binary conversion threshold = 0) and eroded (disk size = 3 pixels). The obtained segmented mask was used to reveal the real vectors. The three resultant vector fields were averaged point by point to generate vector field images. The average tissue velocity for each movie was obtained by averaging the vector norms. Resultant data was plotted using GraphPad Prism software, and two-tailed Mann-Whitney tests were performed between the several conditions (p<0.05). 5 samples were used for each condition.

Proliferation analysis was performed using an ImageJ plugin, ObjectJ. This plugin was used to manually identify: all cytokinesis events, taking into account their orientation, fin fold area and the notochord axis. Resultant data was processed, normalized (area normalization) and corrected (fin fold rotation and translation) in Matlab. Images showing all cell divisions were created using Matlab to project all cell divisions in the correct location relative to the keyframe chosen to represent the movie. Cell division angles were separated into regions according to their location (angle with notochord axis). For each region, angles were divided in 45 degrees intervals, and plotted accordingly. Normalized cell divisions (total numbers) were plotted using GraphPad Prism software, and two-tailed Mann-Whitney tests were performed between the several conditions (p<0.05). For each condition the sample number is n = 5.

Mesenchymal cell movies were registered using the ImageJ plugin MultiStackReg (rigidbody transformation), to correct for non-specific movement.

## Results

### Wound healing is initiated by an abrupt tissue contraction and formation of an actomyosin cable

**Figure 2:**
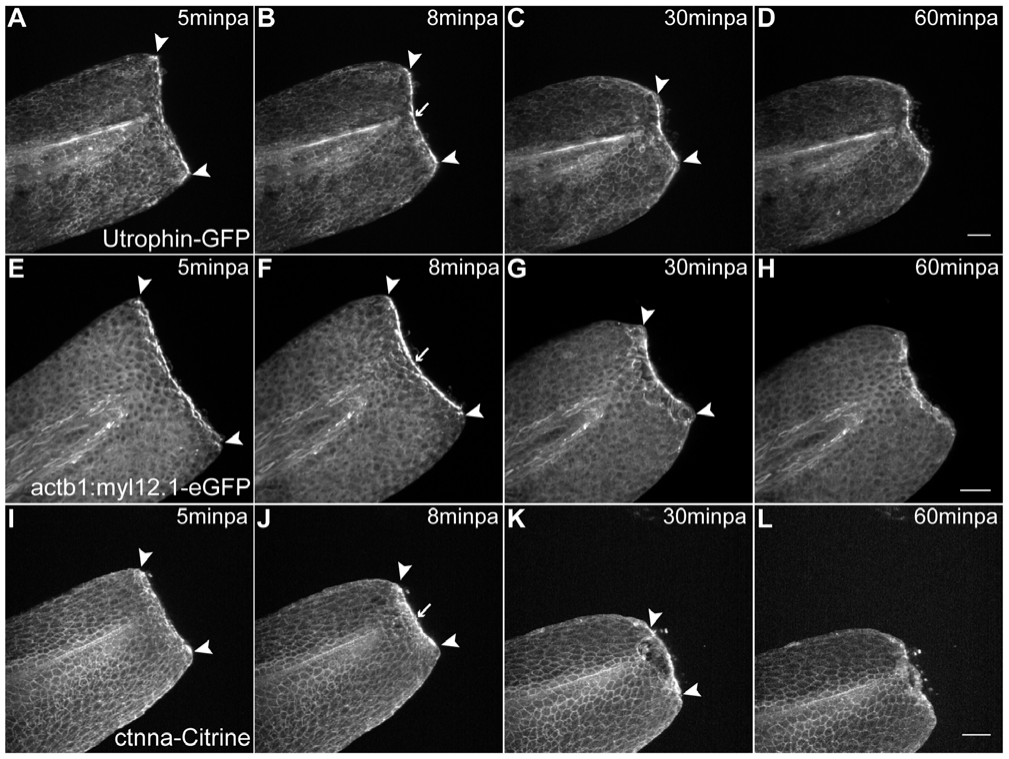
Abrupt tissue contraction and actomyosin cable formation initiate wound healing. **A–D** Sequential images of a representative *in vivo* 55 min time-lapse movie of an Utrophin-GFP mRNA-injected 2 dpf larva at different time-points after amputation. **A** At 5minpa, there is accumulation of actin at the leading edge cells. **B** By 8 minpa the actin cable is assembled (arrow) and there is tissue contraction - note displacement of arrowheads in comparison with A. **C** At 30 minpa, the tissue has fully contracted. **D** At 60 minpa, the wound inflicted by the amputation is closed. **E–H** Sequential images of a representative *in vivo* 55 min time-lapse movie of a 2 dpf *actb1:myl12.1-eGFP* transgenic larva. **E** At 5minpa, there is accumulation of myosin at the leading edge cells. **F** By 8 minpa the myosin cable is assembled (arrow) and there is tissue contraction - note displacement of arrowheads in comparison with E. **G** At 30 minpa, the tissue has fully contracted. **H** At 60 minpa, the wound inflicted by the amputation is closed. **I–L** Sequential images of a representative *in vivo* 55 min time-lapse movie of a 2 dpf *ctnna-Citrine* transgenic larva at different time-points after amputation. **I** At 5minpa, there is accumulation of alpha-catenin at the leading edge cells. **J** By 8 minpa this accumulation appears to localize to the actomyosin cable (arrow) and there is tissue contraction - note displacement of arrowheads in comparison with I. **K** At 30 minpa, the tissue has fully contracted and alpha-catenin is still localized at the wound edge. **L** At 60 minpa, the wound inflicted by the amputation is closed. Anterior is on the left and scale bars correspond to 50 µm in all images; n = 5 larvae per condition.

Upon amputation, the regeneration process is initiated by a wound healing phase. To start the *in vivo* characterization of this process we looked at the dynamics of wound closure upon injury. For that we used live cytoskeleton markers and established a fast time-lapse imaging protocol. In the fin fold, the wound healing is achieved through a rapid contraction of the epidermal tissue through the formation of an actomyosin cable at the leading edge. The accumulation of both actin and myosin is observed as early as 5 minutes post-amputation (minpa), before the main tissue contraction events have happened (Fig. 2A, E, files S1, S2). By 8 minpa the cable is fully formed (Fig. 2B, F arrow) and from this time point until one hour post-amputation (hpa), the tissue contraction exerted by the actomyosin cable appears to be the driving force for the wound to close and to allow the opposite leading edge cells to connect and seal the hole (Fig. 2A–D and E–H, arrowheads, files S1, S2). After the wound is closed (1 hpa), the cable is no longer detected and the tissue relaxes back to its original shape (1–3 hpa) (file S2). Also present at the wound leading edge cells is the adherens junction component, alpha-catenin, which accumulates in a similar manner to actin and myosin (Fig. 2 I–L, file S3). To confirm that this protein co-localizes with the actomyosin cable, we performed immunohistochemistry in 5 minpa alpha-catenin transgenics and observed that indeed alpha-catenin co-localizes with actin in the cable (file S4). This indicates that the actomyosin cable at the leading edge is a complex structure readily assembled to enable contraction of the tissue and sealing of the open wound.

In order to address if the fin fold regeneration dynamics can be affected by the type of injury and/or size of it, we compared the regenerative ability of fin folds with different amputation planes. We could observe that the dynamics were similar in all cases analyzed, namely: in fin folds that were amputated just distal to the notochord (Regular cut); in fin folds that were amputated at a central position between the notochord and the fin tip (Half-size cut); and in fin folds that were diagonally amputated in the dorsal side (Diagonal cut). In all cases the fins recovered at the same time, even though the fins that suffered a Regular cut had to regenerate more tissue in the same period (file S5). Also there were no differences between different zebrafish wild type strains. Since the regeneration dynamics were similar we opted to do a more thorough analysis of the whole process after using always a Regular cut.

### Epidermal tissue growth is increased upon fin fold amputation but maintains its developmental pattern

**Figure 3:**
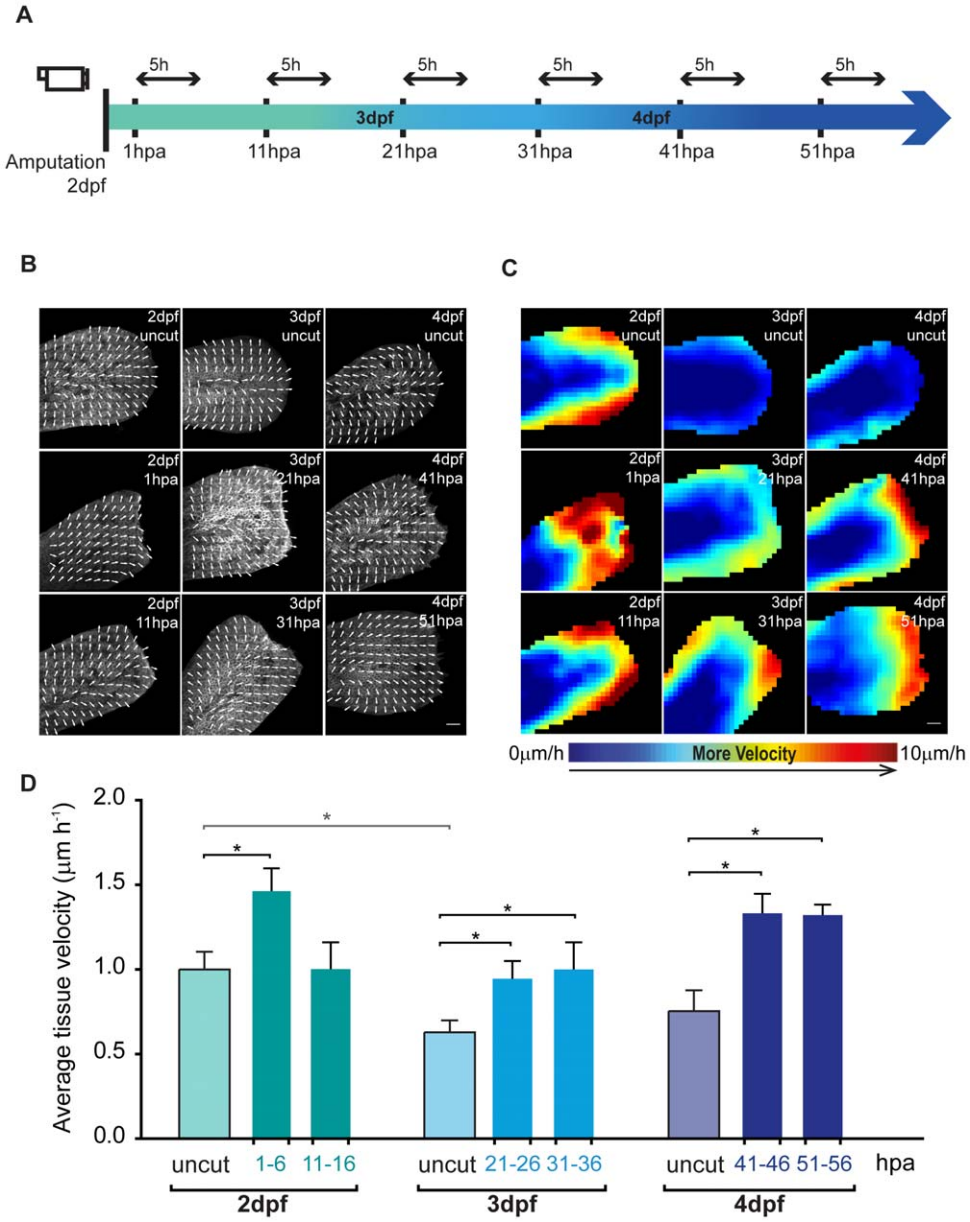
Epidermal tissue growth is enhanced upon fin fold amputation. **A** Experimental outline of the live imaging procedures taking into account not only the post amputation hours (hpa) but also the developmental days of the larvae (dpf). All the amputations were performed in 2 dpf larvae, and these were allowed to regenerate until the desired hour, the time point at which they were imaged for 5 hours. Taking into account that the regeneration procedure takes several days, age-matched uncut controls were imaged in the same conditions and for the same amount of time for accurate comparison. **B** Representative maps of vector velocity fields (VVFs) depicting the tissue movement direction along 5 h sequential time-lapse imaging of the fin fold regenerative process and respective age-matched uncut controls of *β-actin:mGFP* transgenics. **C** Representative heat maps of the VVFs shown in B depicting the tissue velocity in the fin fold area along the same 5 h sequential blocks of the fin fold regenerative process and respective age-matched uncut controls. Red end of the spectrum correlates with higher velocity (0 to 10 µm.hour^−1^) within a given experiment. **D** Average velocity (µm.hour^−1^) of VVFs of 5 larvae per condition represented in B–C. Color code matches the Experimental Outline in A. *P value<0.05; Mann-Whitney test values: 2 dpf uncut<>3 dpf uncut = 0.03; 2 dpf uncut<>1–6 hpa = 0.03; 3 dpf uncut<>21–26 hpa = 0.03; 3 dpf uncut<>31–36 hpa = 0.03; 4 dpf uncut<>41–46 hpa = 0.03; 4 dpf uncut<>51–56 hpa = 0.02; 5 larvae per condition. Anterior is on the left and scale bars correspond to 50 µm.

Following the termination of the wound healing phase, blastema formation takes place and then the fin outgrows. In the zebrafish fin fold, these two last stages are closely interconnected as the fin regenerates in a short period of time. Therefore, we opted to not distinguish between these two phases in very precise time points and instead we considered them as one continuous and progressive process. In order to follow the behavior of epidermal cells, which are the main tissue type that composes the fin fold, we did 5 hour long sequential time-lapse imaging starting at 1 hpa in the fin fold of 2 dpf larvae, using the transgenic *β-actin:mGFP* larvae [19], that labels cell outlines (see Experimental Outline Fig. 3A). We compared the behavior of this tissue upon amputation in 6 time points along the blastema and outgrowth phases with its behavior during normal development in age-matched uncut fins. To quantitatively analyze these data, we performed an image correlation analysis on our images to estimate the velocity fields throughout the fin fold along time. This allowed us not only to see the final position and direction of the displacement vectors in the fin fold epidermis, but also to calculate the rate at which the tissue is expanding. Taking this into account, the general pattern of epidermal growth during development of the fin fold in 2 dpf, 3 dpf and 4 dpf zebrafish larvae is characterized as being radial, expanding distally along the anterior-posterior axis and to the fin's sides (Fig. 3B Uncut). During the blastema and outgrowth phases of regeneration, this pattern is maintained (Fig. 3B), but its average velocity is significantly increased with regard to the age-matched uncut controls, mainly in the distal periphery of the epidermal tissue (Fig. 3C Red Areas). This increase in the average speed in a specific direction of epidermal growth is observed throughout most stages of regeneration when compared to the corresponding uncut developmental stages (Fig. 3D). In summary, the fin fold epidermis has a defined pattern of growth that is not influenced by the occurrence of an amputation. Nevertheless, to recover from amputation, the epidermal tissue responds by significantly increasing its growth rate.

### Proliferation significantly increases during regeneration in a non-circumscribed area

**Figure 4:**
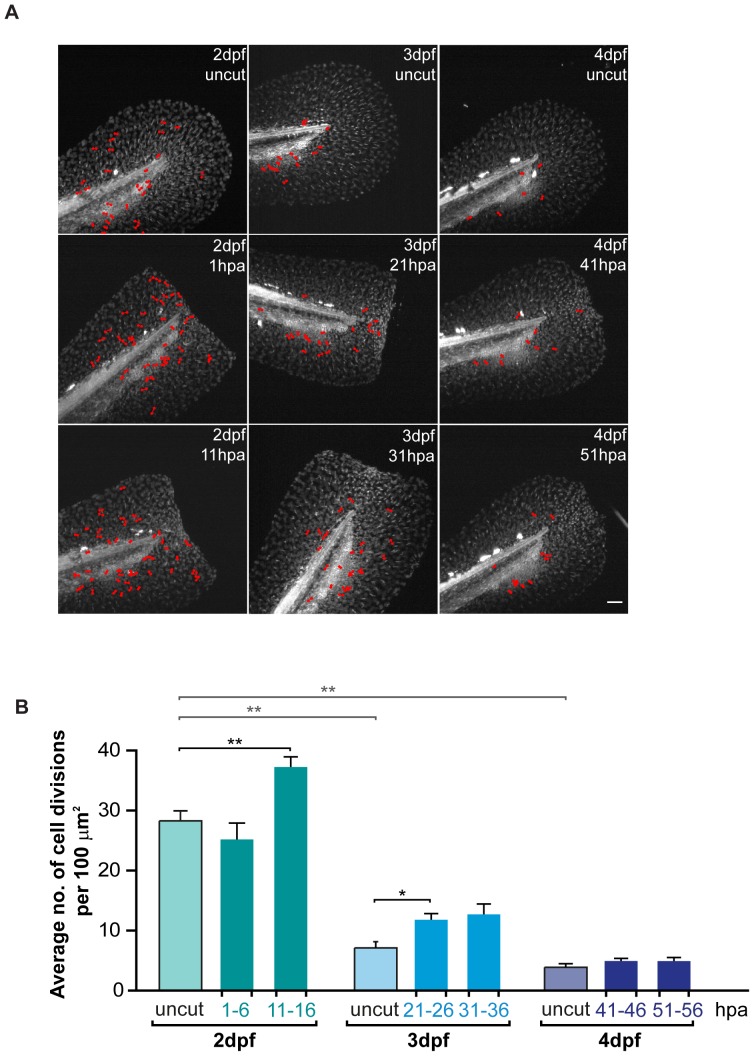
Global levels of proliferation significantly increase during regeneration in a non-spatially restricted manner. **A** Representative projections of *H2a.f/z-GFP* transgenics showing total cell divisions (marked in red) which occurred during 5 hour sequential time-lapse imaging movies of the fin fold regenerative process and respective age-matched uncut controls. **B** Average number of cell divisions occurring per 100 µm^2^ in the several conditions represented in A. Color code matches the Experimental Outline in Fig. 3A. **P value<0.01, *P value<0.05; Mann-Whitney test values: 2 dpf uncut<>3 dpf uncut = 0.008; 2 dpf uncut<>4 dpf uncut = 0.008; 2 dpf uncut<>11–16 hpa = 0.008; 3 dpf uncut<>21–26 hpa = 0.02; 3 dpf uncut<>31–36 hpa = 0.06; 5 larvae per condition. Anterior is on the left and scale bars correspond to 50 µm.

One of the key characteristics of epimorphic regeneration is the occurrence of a boost of proliferation, precisely restricted in time and space mainly in the blastema [28]. To determine whether the observed increase in the velocity of epidermal growth can be due to an increase in proliferation, we performed our *in vivo* time-lapse assay in uncut and amputated fins (Fig. 3A). In order to detect the nuclei of all cells present in the fin fold, we used the transgenic *H2a.f/z-GFP* [20] and quantified all visible cytokinesis events. We found that during larval development, the proliferation rate was progressively reduced from younger (2 dpf) to older (4 dpf) larvae (Fig. 4A–B Uncut). In amputated animals, we observed no differences in the number of cell divisions in the initial stages after wound healing (1–6 hpa), when compared to the 2 dpf uncut control. On the other hand, by 11–16 hpa the cell proliferation had increased significantly when compared to the uncut control (Fig. 4B). At 21–26 hpa, the proliferation continued to be significantly higher with regard to 3 dpf uncut controls, albeit at lower levels than those recorded at 11–16 hpa. From 21 hpa until the end of the regenerative process the proliferation levels kept decreasing, until reaching the same rate as uncut controls (Fig. 4A–B 4 dpf). These results led us to conclude that during regeneration of the fin fold, proliferation is precisely controlled in time. Regarding spatial restriction, we surprisingly observed that the fin fold tissue responded to the amputation by increasing proliferation in a global manner, instead of being restricted to the most distal portion of the fin fold (Fig. 4A), in clear contrast to what happens during zebrafish adult caudal fin regeneration where proliferation is mainly observed within the blastema region [28] [29]. Since proliferation events are many times associated and dependent on apoptosis [30], we performed immunohistochemistry to detect active caspase 3 in uncut and amputated fins throughout the regenerative process and could never find significant levels of apoptosis in both experimental conditions (File S6). In conclusion, the zebrafish fin fold shows a timely control of proliferation upon amputation, but this is not delimited to a region of the fin fold, happening generally throughout the fin.

### Cell divisions in the fin fold are stereotypically oriented during normal development and follow a randomization tendency upon amputation

**Figure 5:**
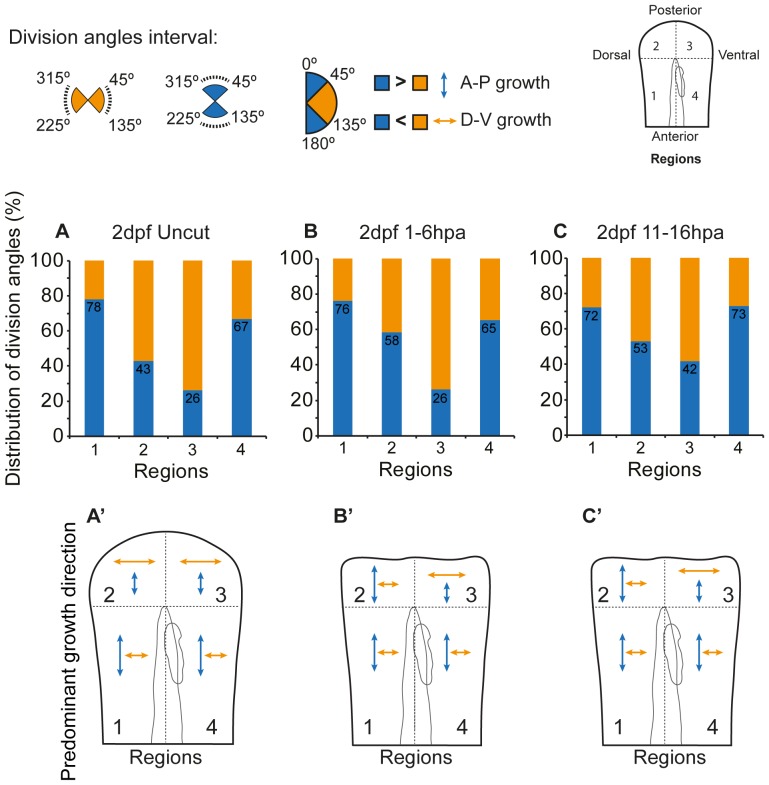
Cell division orientation in the fin fold shows a tendency for randomization predisposition upon amputation. Distribution of division angles according to the interval of 225–315° and 45–135° (orange) or 315–45° and 135–225° (blue) in the designated regions 1–4 of the fin fold in **A** 2 dpf uncut control **B** 2 dpf 1–6 hpa **C** 2 dpf 11–16 hpa. Predominant growth direction in the regions 1–4 taking into account the orientation of the majority of cell divisions happening in **A'** 2 dpf uncut control **B'** 2 dpf 1–6 hpa **C'** 2 dpf 11–16 hpa. n = 193 divisions in 2 dpf uncut, n = 175 divisions in 2 dpf 1–6 hpa, n = 258 divisions in 2 dpf 11–16 hpa; 5 larvae per condition.

It has been shown that the orientation of cell division is essential during embryonic development and contributes throughout growth and patterning events of several structures as well as during elongation of the embryo [31] [23]. Since we observed significant changes in the cell proliferation rate upon amputation, we asked whether the orientation of cell division could contribute to the regeneration of the fin fold. To analyze this, we divided the fin fold in 4 regions by establishing 2 main axes, one parallel along the notochord (anterior-posterior axis) and one just distal to the tip of the notochord, perpendicular to it (dorso-ventral axis) (Fig. 5). This allowed us to have a reference point to measure the angles at which cytokinesis occurs, in a 360 degree scale. In 2 dpf uncut control larvae, in regions 1 and 4, which are laterally located to the notochord, 78% and 67% of all cell divisions observed occurred in the anterior-posterior (A-P) axis, respectively (Fig. 5A). These had a predominant angle of cytokinesis occurring mainly in the intervals of 315–45 degrees and 135–225 degrees (Fig. 5A and file S6). Hence the major direction of growth in these regions is parallel to the notochord, which likely contributes to the elongation of the body axis (Fig. 5A'). Regarding regions 2 and 3, distally located to the notochord, 57% and 74% of the cell divisions occurred in the dorso-ventral (D-V) axis respectively, since they had a predominant angle of cytokinesis in the interval of 225–315 degrees and 45–135 degrees (Fig. 5A). This pattern of growth likely allows the fin fold to expand to its sides (Fig. 5A'). These results are in accordance with the pattern of growth obtained in our vector velocity field analysis (Fig. 3B) and led us to conclude that the cell divisions in the zebrafish fin fold are stereotypically oriented during its development. A more detailed analysis using 45 degree angle intervals led to the same conclusions (file S7).

Upon amputation, no statistically significant differences were observed in any of the analyzed regions when compared to uncut controls. Cell divisions happening in regions 1 and 4 kept the same behavior as in uncut controls, preserving the stereotypical orientations (Fig. 5B–C; B'–C'; Regions 1 and 4); nevertheless, we verified a slight increase in the percentage of cell divisions in the A-P axis in regions 2 and 3 when compared to the same regions in uncut controls, both in the first regeneration time point (Fig. 5B) as well as in the proliferation peak time point (Fig. 5C). Thus, there seems to be a tendency for randomization of the angles of cell division during these regeneration stages (Fig. 5B'–C'). During later stages of regeneration, we could not establish this analysis due to low cell division numbers. In summary, we conclude that changes in the pattern of cell division orientation do not seem to be a major determinant of fin growth during regeneration.

### Mesenchymal cells alter their shape and migrate distally upon amputation

**Figure 6:**
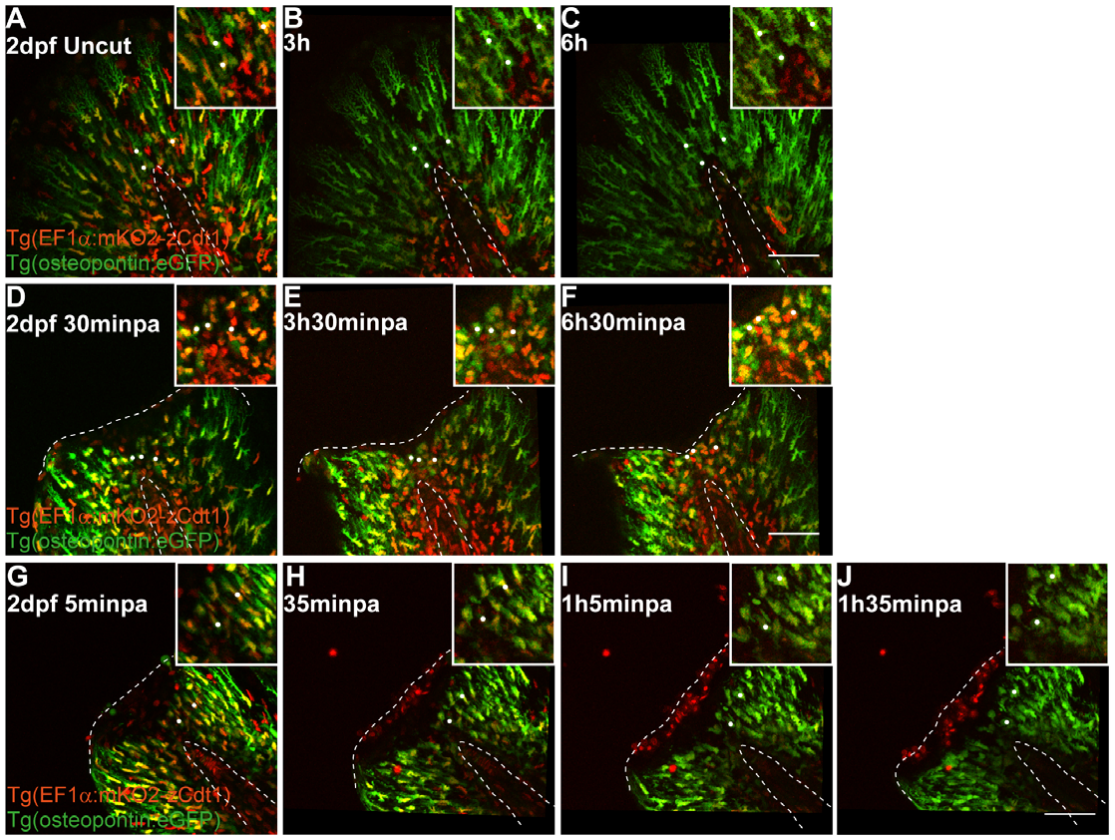
Mesenchymal cells in the fin fold change shape and migrate distally upon injury. **A–C** Sequential images of a representative *in vivo* 6 h time-lapse movie of a 2 dpf uncut *EF1α:mKO2-zCdt1;osteopontin:eGFP* transgenic larva. **D–F** Sequential images of a representative *in vivo* 6 h time-lapse movie of a 2 dpf 30 minpa *EF1α:mKO2-zCdt1;osteopontin:eGFP* transgenic larva. **G–J** Sequential images of a representative *in vivo* 1h30 time-lapse movie of a 2 dpf 5 minpa *EF1α:mKO2-zCdt1;osteopontin:eGFP* transgenic larva. Zoom panels highlight *osteopontin* positive mesenchymal cells in the central area of the fin fold in the respective time point. White dots mark the same cells along time to allow for better visualization and tracking of cell migration. Dashed lines indicate the outline of the notochord and the edge of the amputated fin fold. Scale bars correspond to 50 µm in all images; 3–5 larvae per condition.

Besides the epidermis, the fin fold is composed of mesenchymal cells. It has been suggested that these cells give rise to the blastema upon amputation, like in the adult fin system [32], although to our knowledge there is no detailed *in vivo* characterization of these cells during regeneration of the fin fold. These mesenchymal cells are fibroblast-like with a very particular shape, being very elongated with extended protrusions and stretched nuclei [13] [16]. It is also known that they are migratory, and play a role in providing stabilization and structure to the fin fold by secreting actinotrichia [13] [16] [14]. To understand the function of these cells during regeneration, we used a transgenic that expresses GFP driven by the *osteopontin* promoter. This transgenic labels numerous structures in the zebrafish, including the mesenchymal cells in the fin fold (file S8). To follow and track the migration of these cells, we performed time-lapse imaging (up to 12 hour long) of double positive larvae for *osteopontin:eGFP* and for the cell cycle nuclear marker *EF1α:mKO2-zCdt1* (see Methods). We found that during development at 2 dpf, the *osteopontin*-positive mesenchymal cells do not appear to migrate (Fig. 6A–C, file S9). However, upon amputation, as early as 30 minpa, a fraction of the mesenchymal cells actively migrated towards the injury (Fig. 6D–F, file S10). The migrating cells were originally located radially around the notochord, in the center of the fin fold. In addition to migrating, these cells lost their original elongated shape and acquired a more rounded form (Fig. 6D–F, Zoom panels, white dots), accumulating distally to the notochord in a blastemal-like zone. Of note, the *osteopontin-eGFP* positive mesenchymal cells always maintained the *mKO2-zCdt1* labeling during the *in vivo* imaging (Fig. 6) and throughout the next regenerating days (file S11). The protein zCdt1 is a marker for the G0-G1 phase of the cell cycle [22], therefore our results indicate that these cells are kept in this phase of the cell cycle, even upon amputation. These data allow us to conclude that the observed changes in cell shape are not directly linked to cell proliferation.

To characterize further the spatiotemporal dynamics leading to small morphology changes in the mesenchymal cells at a single cell level, we performed faster and shorter (up to 3 h with acquisition every minute) time-lapse imaging of the same transgenic *EF1α:mKO2-zCdt1;osteopontin:eGFP*, starting at 5 minutes after amputation. In fact, by monitoring the mesenchymal cells in this manner we could detect the initial loss of elongation and rounding up of these cells as early as 35 minpa, and until 1h35 minpa this was a progressive change of morphology (Fig. 6G–J Zoom panels, white dots). These changes happened at the same time as the wound healing process was still taking place, indicating a quick reaction of the mesenchymal cells upon amputation and coincided with the onset of distal migration of these cells. To clarify further the correlation between the migration and the change of cell shape of the mesenchymal cells, we accessed their polarization state. In migrating mesenchymal cells, centrosomes typically assume a position between the leading edge and the nucleus [33] [34]. To address this in our system, we compared the location of the centrosome/microtubule organizing center (MTOC) relative to the cell nucleus [34], by performing gamma-tubulin (γTubulin) immunohistochemistry in *osteopontin:eGFP* transgenic animals. We found that in 2 dpf uncut fin folds, the *osteopontin* positive mesenchymal cells had their MTOCs positioned between the nucleus and the stretched protrusions, and in many of these cells, the distance between the MTOC and the nucleus was remarkable (File S12, A–A' Uncut). Upon amputation, during the cell shape change and active migration time-points (1 hpa), the MTOC position was maintained between the nucleus and leading edge of the cells, but in close proximity to the nucleus, indicating that these cells did not lose their polarization state during these events (File S12, B–B' 1 hpa). By 1 day post-amputation (dpa), when the distal migration and change of shape events are completely concluded, the MTOC position was variable (File S12, C–C' 1 dpa): in some cells the MTOC was positioned still between the nucleus and leading edge while in others the MTOC assumed a position in the rear of the cell. This indicates that the polarization of mesenchymal cells can be lost after the complete accumulation in the blastemal-like zone.

**Figure 7:**
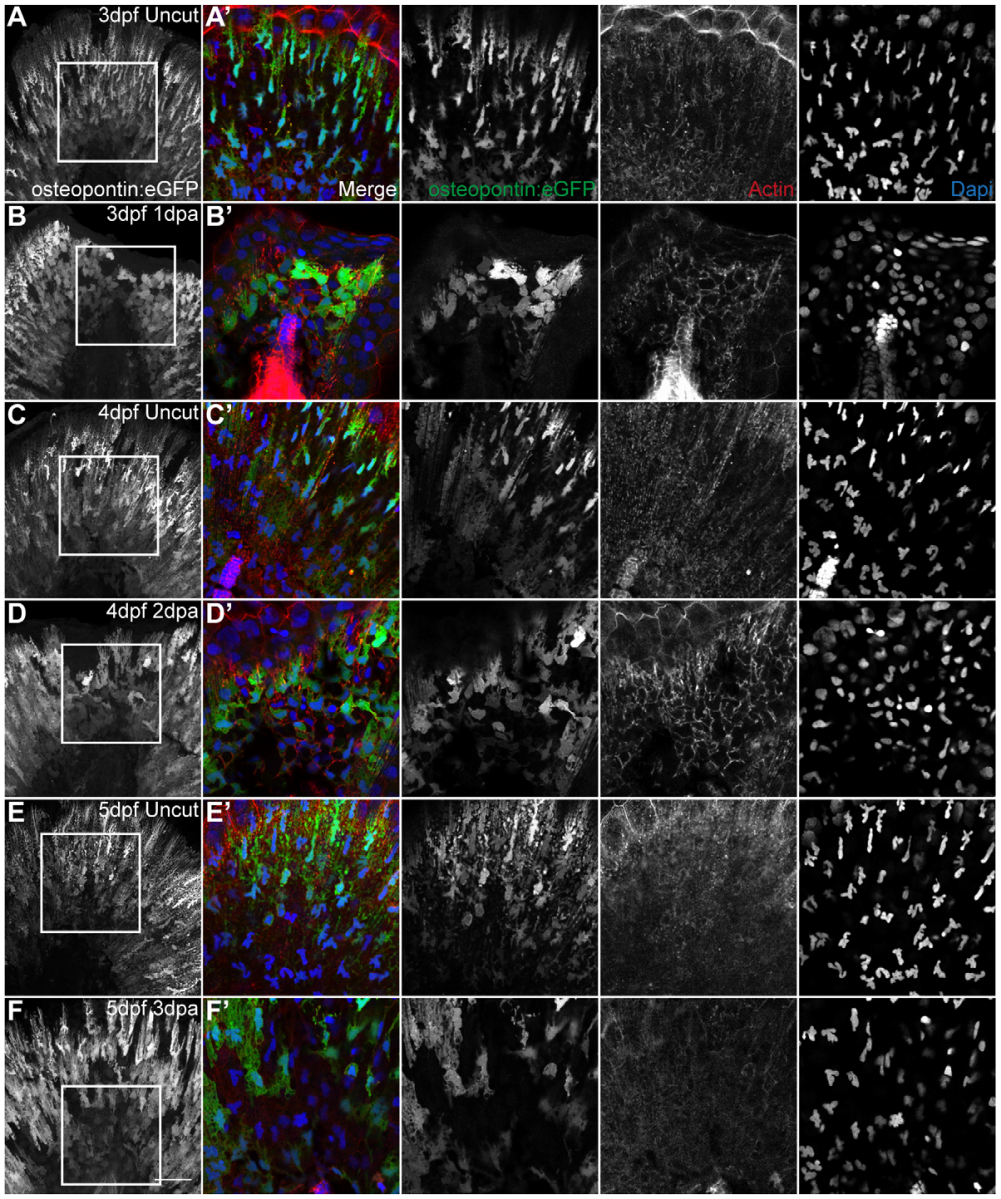
The shape modification of mesenchymal cells lasts throughout and is specific of regeneration. **A,C,E** Representative immunostaining with anti-GFP antibody in uncut transgenic *osteopontin:eGFP* larvae of 3 dpf, 4 dpf and 5 dpf respectively. **B,D,F** Representative immunostaining with anti-GFP antibody in amputated transgenic *osteopontin:eGFP* larvae of 3 dpf 1 dpa, 4 dpf 2 dpa and 5 dpf 3 dpa respectively. **A–F** Representative z-stack projections of the *osteopontin:eGFP* labeling. **A'–F'** Representative single frames of the corresponding zoomed area represented by a square in A–F. Merged and single color images of *osteopontin:eGFP* labeling the mesenchymal cells (anti-GFP, green), actin (phalloidin, red) and nuclei (DAPI, blue), respectively. 5 Larvae per condition. Scale bar corresponds to 50 µm in all images.

To address whether the cell shape changes in mesenchymal cells would continue throughout the rest of the regenerative process, we did immunohistochemistry in the *osteopontin:eGFP* transgenic fish. We confirmed that at 1 and 2 dpa, groups of mesenchymal cells had accumulated near the amputation plane and had become rounder both in terms of cytoplasm and nucleus, when compared with uncut controls (Fig. 7A–D *osteopontin:eGFP* and Dapi). In addition, the cortical actin present in the regenerating fin fold had developed a complex meshwork in the round cells (Fig. 7B,D Actin). Both of these features were undetected at 3 dpa and cell shapes resembled the uncut control, indicating that these events were specific to the regeneration process (Fig. 7E–F).

## Discussion

In this work we have systematically characterized the fin fold regeneration process through *in vivo* studies. We analyzed the wound closure dynamics, the contributions of epidermal growth, proliferation levels and orientation of cell divisions necessary to achieve a functional and proper sized fin fold. Also, we have explored the role of mesenchymal cells in this process.

Our findings show that during zebrafish fin fold regeneration there is an actomyosin based mechanism used for wound closure, which seems to be conserved in many other organisms [35] [36] [37]. This process is extremely rapid and involves major tissue contraction and relaxation. Importantly, the co-localization of the adherens junctions component alpha-catenin with the actomyosin cable raises interesting questions about the complexity and function of this essential structure. This is in accordance with the presence of another adherens junctions marker, beta-catenin, at the leading edge cells during wound healing of the zebrafish fin fold [11].

During fin development, the epidermis has a precise pattern of movement and growth that is maintained in the blastema and outgrowth phases of regeneration. On the other hand, during regeneration, the growth rate is significantly increased in all time points apart from 11–16 hpa, to recover the fin's original shape and size in a timely manner. Interestingly, there is a significant increase in proliferation in response to the amputation, which happens precisely at 11–16 hpa. This indicates that, like in the adult caudal fin regeneration system, the proliferation is controlled in time [28]. However, in contrast to the adult system, this increase in proliferation does not seem to be spatially restricted [28] [29]. In fact, during the proliferation peak, we noticed cytokinesis events happening in a broad area of the fin fold, and not only in the most distal tissue. It appears that the fin fold blastema does not have a specific function for proliferation and in that way it is not a classical blastema, as observed in the adult system.

Previous reports have shown that proliferation of blastema-like cells increases upon amputation of the larval fin fold, however, the authors suggested that such proliferation is spatially restricted [9] [10] [11]. This work was based on BrdU pulse-chase experiments where cells were labeled at different time points after amputation and their position within the fin fold accessed at the end of the regeneration process. In contrast our data shows that in the first hours after amputation the number of cell divisions increases but not in a preferential region of the fin. We propose that this discrepancy might be due to the distinct methods used to determine proliferation in the two studies. We believe that our characterization of the full process *in vivo* clearly reveals a global proliferation response, a property that appears to be specific of fin fold regeneration.

When comparing the epidermal growth and proliferation events, we observed that the tissue growth rate seems to increase with time during the regeneration process at the same time as proliferation slows down (compare Fig. 3D and 4B), implying that tissue growth is compensated by a mechanism that does not depend on proliferation. The only time point at which the velocity of epidermal growth is not significantly increased is when the proliferation is at its peak (between 11 and 16 hpa), suggesting that there are other outgrowth mechanisms that contribute crucially to this process.

We show, for the first time, that the mesenchymal cells present in the fin fold react to amputation by migrating and accumulating distally. This is accompanied by a change of cell shape and an increase in the complexity of the cortical network of actin that is assembled in these cells. In fact, previous reports addressing these cells during fin fold regeneration, show that there is an increase in the condensation of their nuclei [9]. This is in accordance with our cell shape change results, where we found a clear transformation of the mesenchymal cells typical shape into a more round form, including in their nuclear shape. This alteration in shape could be due to these cells entering an active cell cycle process, initiating cell division; however this does not seem to be the case, since the mesenchymal cells remain in the G0–G1 phase of the cell cycle throughout the full regeneration process. It is also possible that this change in morphology is part of a dedifferentiation process triggered by the amputation, as in the adult caudal fin model where these cells acquire a round form and migrate distally to constitute the blastema [4] [5]; nevertheless the differentiation state of the fin fold mesenchymal cells is still uncharacterized. There are indications that during the fin fold development, these cells acquire progressively their protrusive morphology, suggesting that this is consistent with a more differentiated status [13] [14]; if that is the case, then the mesenchymal cells may be undergoing dedifferentiation during the fin fold's regenerative process. The function of these cells in the regeneration process is still unclear, but it is possible that they are part of a signaling center [10] [12] [38], and to that extent, constitute the so-called blastema in a comparable manner to the adult system.

## Conclusions

Altogether this study presents a global view and a better understanding of how the regenerative process is accomplished in the larvae fin fold. We performed a systematic analysis of the regenerative response of the main cell types present in this structure and characterized in detail several mechanisms by which the fin regenerates. With this work we show for the first time the *in vivo* dynamics of wound closure during the initial phases of the regeneration process; we illustrate the existence of a developmental pattern of growth of the fin fold, which is maintained upon amputation, albeit with a significantly increased rate of growth; we demonstrate that the cell proliferation response to damage in epidermal cells occurs in a broad region away from the wound, and lastly, we show that the mesenchymal cells are highly polarized and undergo dramatic cell shape changes during the regeneration process. These findings provide novel insight into the *in vivo* mechanisms occurring during the fin fold regeneration process, contributing to a better knowledge of this regenerative system and making it a valuable model to investigate further questions in the regeneration field.

## Supporting Information

File S1 **Wound healing process of 2 dpf Utrophin-GFP injected fish.** 1 hour movie of a 2 dpf 5 minpa larva. Anterior is to the left. Scale bar corresponds to 50 µm.
(MOV)

File S2 **Wound healing process of 2 dpf** ***actb1:myl12.1-eGFP*** **transgenic fish.** 3 hours movie of a 2 dpf 5 minpa larva. Anterior is to the left. Scale bar corresponds to 50 µm.
(MOV)

File S3 **Wound healing process of 2 dpf** ***Alpha-Catenin-Citrine*** **transgenic fish.** 1 hour movie of a 2 dpf 5 minpa larva. Anterior is to the left. Scale bar corresponds to 50 µm.
(MOV)

File S4 **Alpha-Catenin is present in the actin cable during wound healing.** 3D reconstruction of a representative immunostaining against anti-GFP antibody to detect alpha-catenin (green) and phalloidin to detect actin (red) in 2 dpf 5 minpa *alpha-catenin-Citrine* (*ctnna-Citrine*) transgenic larva. n = 5 larvae.
(MOV)

File S5 **The fin fold regeneration dynamics are independent of the size of amputation.** Representative brightfield live images of AB (**A–L**) and TU (**M–X**) larvae during several stages of the regenerative process and their respective age-matched uncut controls. Larvae were subjected to different amputation planes (Regular, Half-size and Diagonal cuts) and followed throughout the next 3 regenerating days, time point in which the regenerative ability was accessed. 5 Larvae per condition.
(TIF)

File S6 **Apoptosis is not present during fin fold regeneration.** Representative immunofluorescence with anti-active Caspase3 antibody in uncut and amputated larvae of 3 dpf (**A–B**), 4 dpf (**C–D**) and 5 dpf (**E–F**). Arrow indicates the presence of an apoptotic cell. n = 5 larvae per condition. Scale bar corresponds to 50 µm in all images.
(TIF)

File S7 **Detailed distribution of cell division angles in the 4 regions of the fin fold.**
(TIF)

File S8 **Expression pattern of the 2 dpf transgenic** ***osteopontin:eGFP*** **.** At this stage of development, this transgenic has labeled the fin fold mesenchymal cells, but also other mesenchymal cells that are spread out along the midline and somites. Besides these, the pectoral fin, the eye and the brain are also GFP positive.
(TIF)

File S9 **2 dpf Uncut control double transgenic** ***EF1α:mKO2-zCdt1;osteopontin:eGFP*** **.** 6 hours movie of an uncut 2 dpf larva. Scale bar corresponds to 50 µm.
(MOV)

File S10 **2 dpf 30 minpa double transgenic** ***EF1α:mKO2-zCdt1;osteopontin:eGFP*** **. 6 hours movie of a 2 dpf larva 30 minpa.** Scale bar corresponds to 50 µm.
(MOV)

File S11 **The mesenchymal cells are maintained in G0-G1 phases of the cell cycle regardless of an amputation.** Live imaging representative images of double transgenic *EF1α:mKO2-zCdt1;osteopontin:eGFP* larvae during several stages of the regenerative process and their respective controls. **A,C,E** are uncut (3 dpf, 4 dpf and 5 dpf respectively) and age matched controls for **B,D,F** (3 dpf 1 dpa, 4 dpf 2 dpa, 5 dpf 3 dpa respectively). Merged and single color images corresponding to *osteopontin:eGFP* labeling the cytoplasm of the mesenchymal cells (green) and *mKO2-zCdt1* labeling the nuclei of fin fold cells in G0–G1 phases of the cell cycle (red). 3 larvae per condition. Scale bar corresponds to 50 µm.
(TIF)

File S12 **The mesenchymal cells are polarized.** **A** Representative immunostaining with anti-GFP and anti-γTubulin antibodies in 2 dpf uncut transgenic *osteopontin:eGFP* larvae (single frame). **B–C** Representative immunostaining with anti-GFP anti-γTubulin antibodies in amputated transgenic *osteopontin:eGFP* larvae of 2 dpf 1 hpa and 3 dpf 1 dpa (single frames). **A'–C'** Representative single frames of the corresponding zoomed area represented by a square in A–C. Merged and single color images of the MTOC (γTubulin, red) together with the nuclei (DAPI, blue) and *osteopontin:eGFP* labeling the mesenchymal cells (anti-GFP, green), respectively. The arrows highlight the presence of a MTOC to allow better comparison of its position relative to the corresponding nucleus. 5 Larvae per condition. Scale bar corresponds to 50 µm in all images.
(TIF)
