# Supplementary figures and images for: In Vivo Cell and Tissue Dynamics Underlying Zebrafish Fin Fold Regeneration
Source: PLoS One. 2012 Dec 20;7(12):e51766. doi: 10.1371/journal.pone.0051766 (PMC3527495; doi:10.1371/journal.pone.0051766)

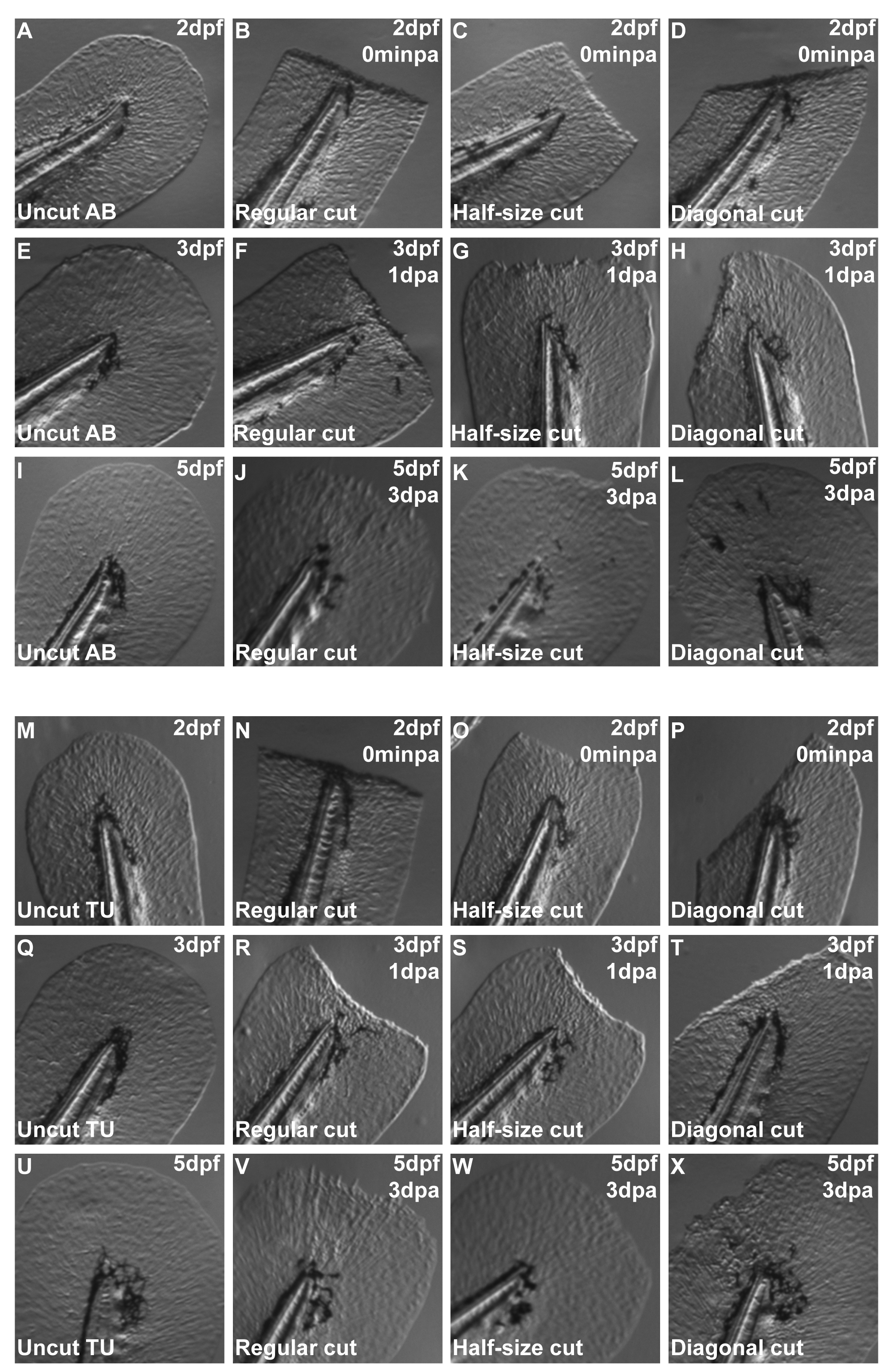

Supplement: File S5 — The fin fold regeneration dynamics are independent of the size of amputation. Representative brightfield live images of AB (A–L) and TU (M–X) larvae during several stages of the regenerative process and their respective age-matched uncut controls. Larvae were subjected to different amputation planes (Regular, Half-size and Diagonal cuts) and followed throughout the next 3 regenerating days, time point in which the regenerative ability was accessed. 5 Larvae per condition. (TIF) [file pone.0051766.s005.tif]

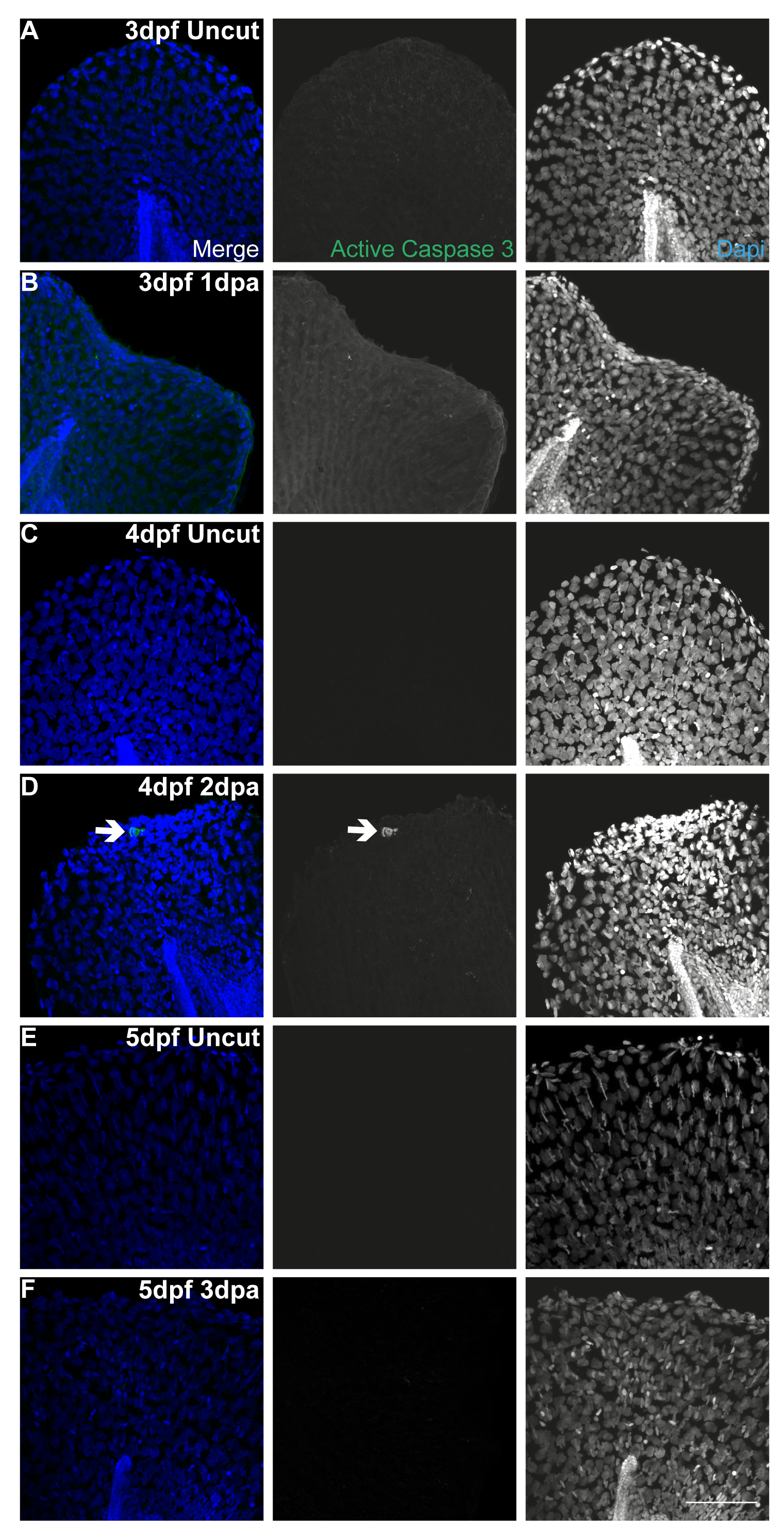

Supplement: File S6 — Apoptosis is not present during fin fold regeneration. Representative immunofluorescence with anti-active Caspase3 antibody in uncut and amputated larvae of 3 dpf (A–B), 4 dpf (C–D) and 5 dpf (E–F). Arrow indicates the presence of an apoptotic cell. n = 5 larvae per condition. Scale bar corresponds to 50 µm in all images. (TIF) [file pone.0051766.s006.tif]

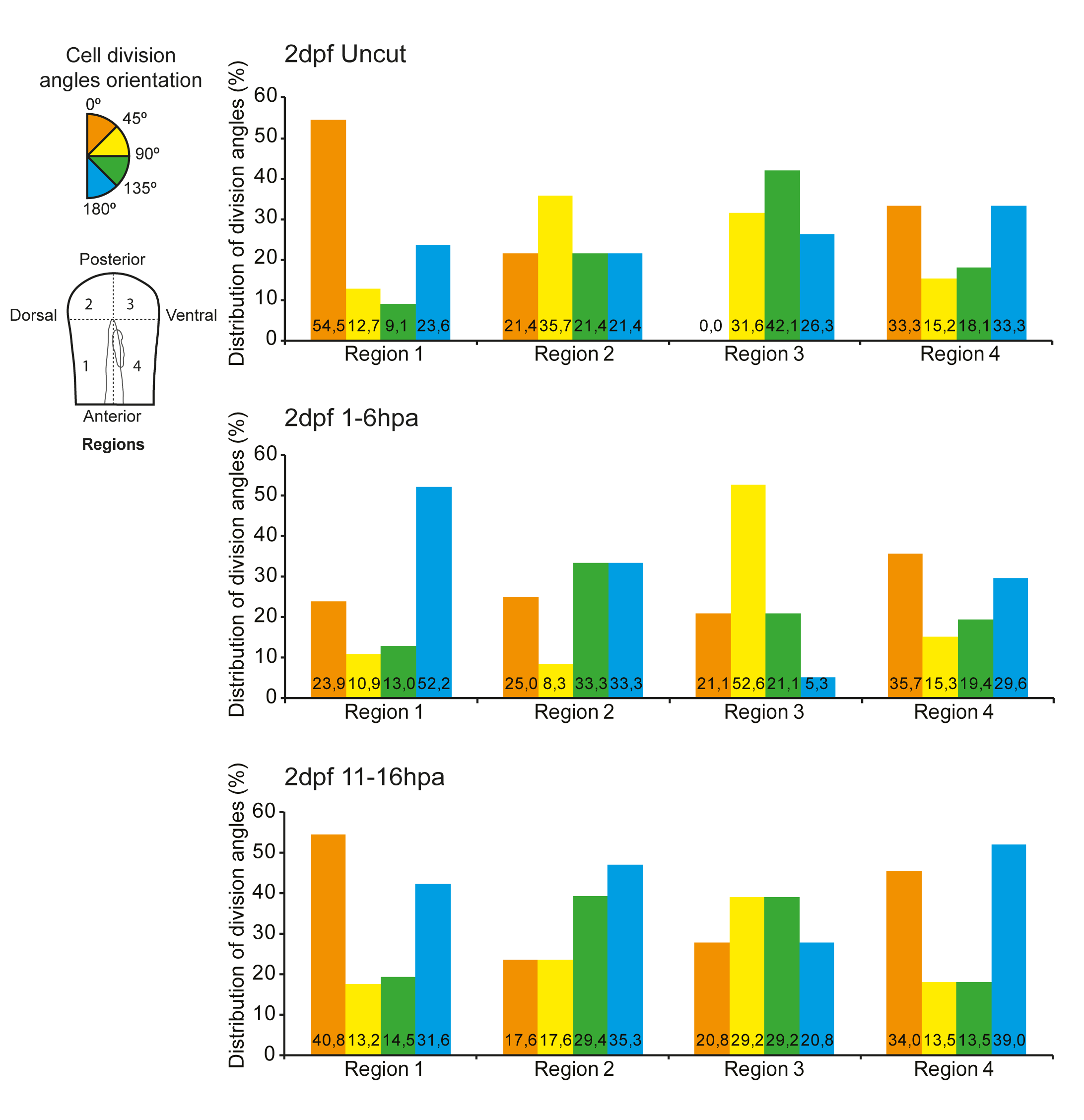

Supplement: File S7 — Detailed distribution of cell division angles in the 4 regions of the fin fold. (TIF) [file pone.0051766.s007.tif]

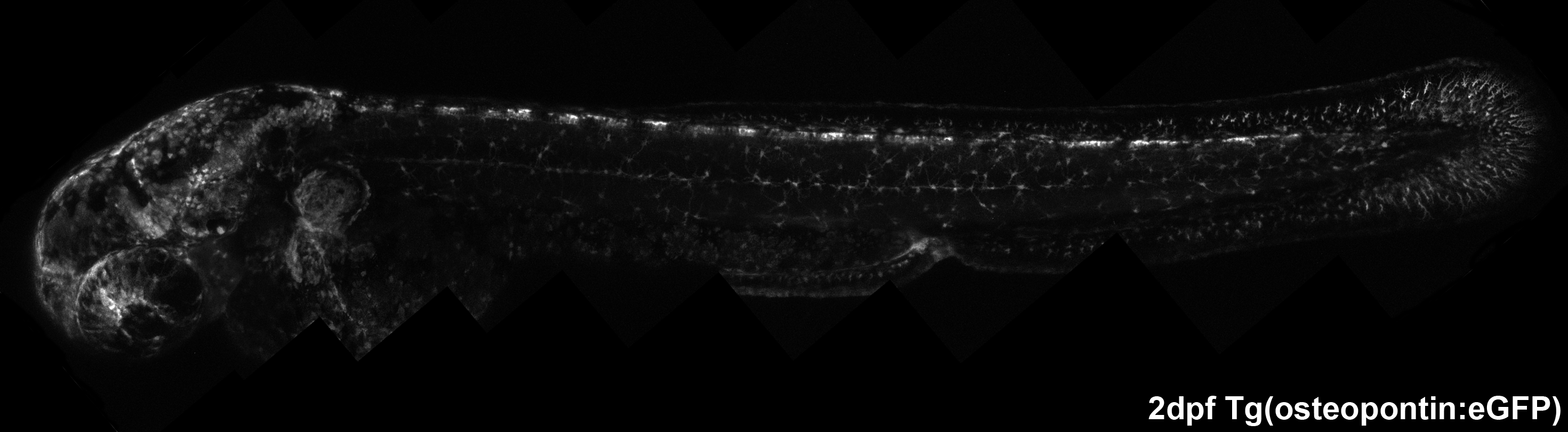

Supplement: File S8 — Expression pattern of the 2 dpf transgenic osteopontin:eGFP . At this stage of development, this transgenic has labeled the fin fold mesenchymal cells, but also other mesenchymal cells that are spread out along the midline and somites. Besides these, the pectoral fin, the eye and the brain are also GFP positive. (TIF) [file pone.0051766.s008.tif]

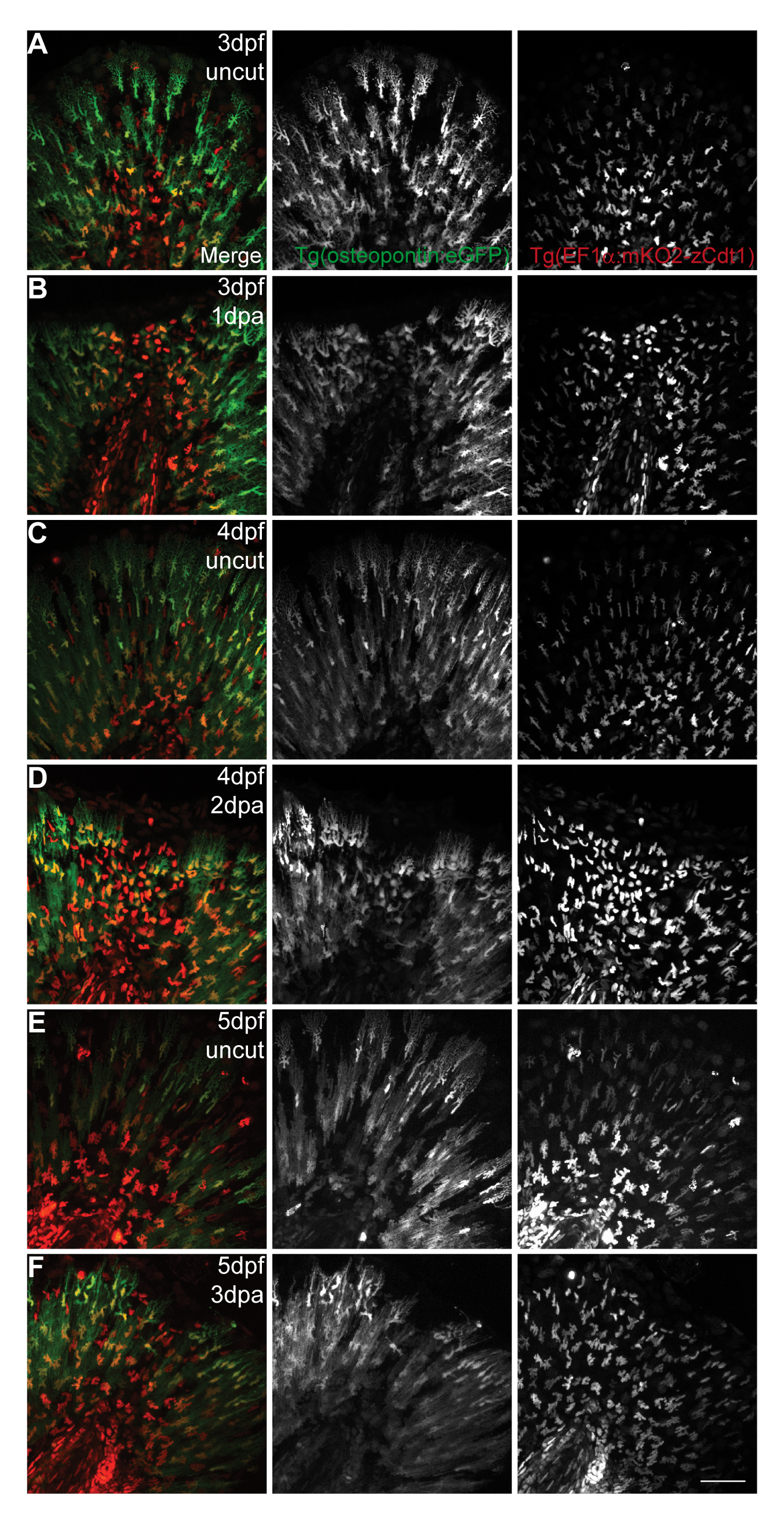

Supplement: File S11 — The mesenchymal cells are maintained in G0-G1 phases of the cell cycle regardless of an amputation. Live imaging representative images of double transgenic EF1α:mKO2-zCdt1;osteopontin:eGFP larvae during several stages of the regenerative process and their respective controls. A,C,E are uncut (3 dpf, 4 dpf and 5 dpf respectively) and age matched controls for B,D,F (3 dpf 1 dpa, 4 dpf 2 dpa, 5 dpf 3 dpa respectively). Merged and single color images corresponding to osteopontin:eGFP labeling the cytoplasm of the mesenchymal cells (green) and mKO2-zCdt1 labeling the nuclei of fin fold cells in G0–G1 phases of the cell cycle (red). 3 larvae per condition. Scale bar corresponds to 50 µm. (TIF) [file pone.0051766.s011.tif]

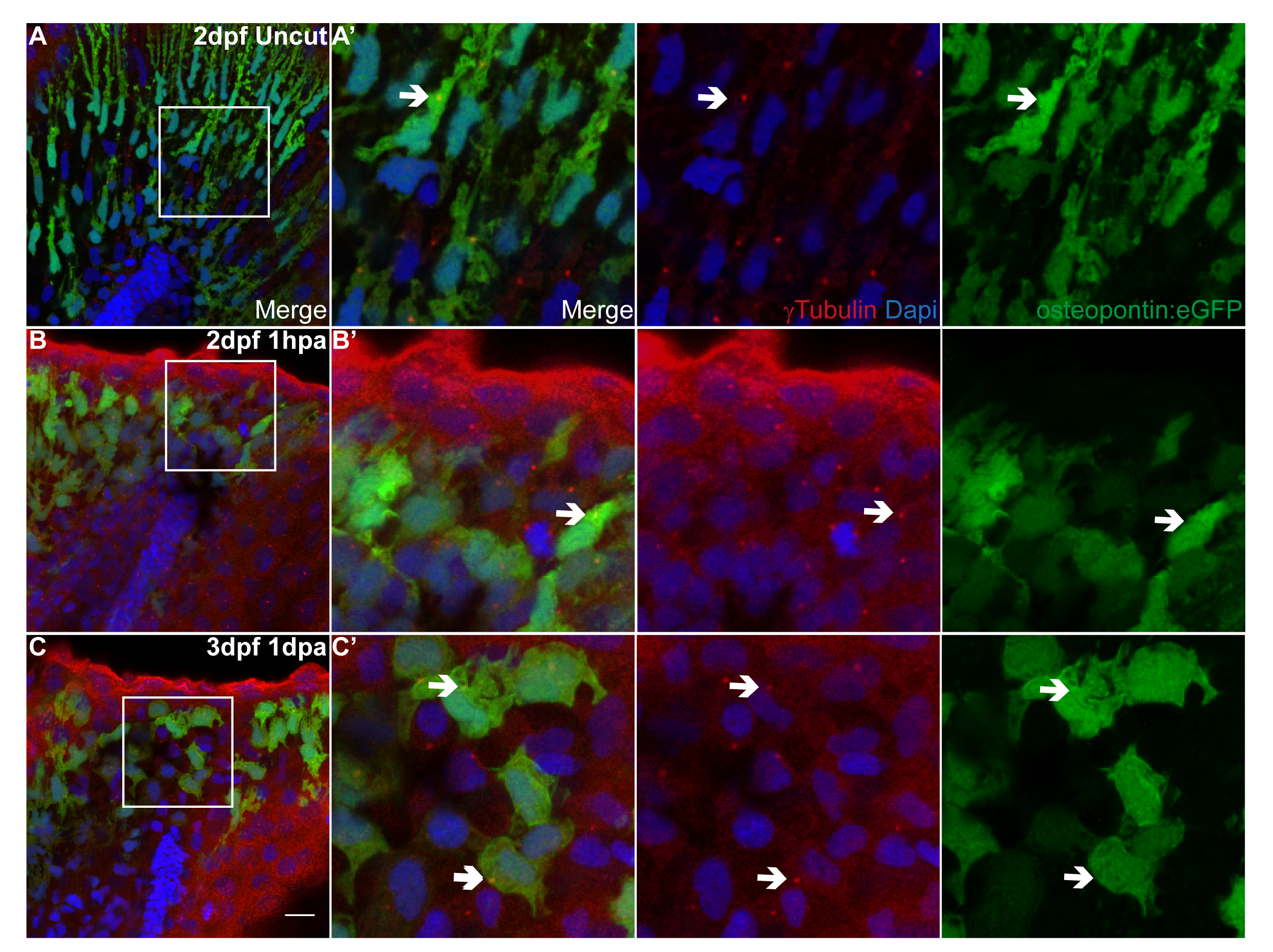

Supplement: File S12 — The mesenchymal cells are polarized. A Representative immunostaining with anti-GFP and anti-γTubulin antibodies in 2 dpf uncut transgenic osteopontin:eGFP larvae (single frame). B–C Representative immunostaining with anti-GFP anti-γTubulin antibodies in amputated transgenic osteopontin:eGFP larvae of 2 dpf 1 hpa and 3 dpf 1 dpa (single frames). A'–C' Representative single frames of the corresponding zoomed area represented by a square in A–C. Merged and single color images of the MTOC (γTubulin, red) together with the nuclei (DAPI, blue) and osteopontin:eGFP labeling the mesenchymal cells (anti-GFP, green), respectively. The arrows highlight the presence of a MTOC to allow better comparison of its position relative to the corresponding nucleus. 5 Larvae per condition. Scale bar corresponds to 50 µm in all images. (TIF) [file pone.0051766.s012.tif]
